# Oxidants, antioxidants and the current incurability of metastatic cancers

**DOI:** 10.1098/rsob.120144

**Published:** 2013-01

**Authors:** Jim Watson

**Affiliations:** Cold Spring Harbor Laboratory, Cold Spring Harbor, New York, NY 11724, USA

**Keywords:** cancer, reactive oxygen species, metastatic cancer

## Abstract

The vast majority of all agents used to directly kill cancer cells (ionizing radiation, most chemotherapeutic agents and some targeted therapies) work through either directly or indirectly generating reactive oxygen species that block key steps in the cell cycle. As mesenchymal cancers evolve from their epithelial cell progenitors, they almost inevitably possess much-heightened amounts of antioxidants that effectively block otherwise highly effective oxidant therapies. Also key to better understanding is why and how the anti-diabetic drug metformin (the world's most prescribed pharmaceutical product) preferentially kills oxidant-deficient mesenchymal p53^− −^cells. A much faster timetable should be adopted towards developing more new drugs effective against p53^− −^ cancers.

Although the mortality from many cancers, particularly those of haematopoietic cells, has been steadily falling, the more important statistic may be that so many epithelial cancers (carcinomas) and effectively all mesenchymal cancers (sarcomas) remain largely incurable. Even though an increasing variety of intelligently designed, gene-targeted drugs now are in clinical use, they generally only temporarily hold back the fatal ravages of major cancers such as those of the lung, colon and breast that have become metastatic and gone beyond the reach of the skilled surgeon or radiotherapist. Even though we will soon have comprehensive views of how most cancers arise and function at the genetic and biochemical level, their ‘curing’ seems now to many seasoned scientists an even more daunting objective than when the ‘War on Cancer’ was started by President Nixon in December 1971.

Propelling me then, 40 years ago, to turn the Cold Spring Harbor Laboratory into a major site for unravelling the genetic underpinnings of cancer was the belief that once the gene-induced molecular pathways to cancer became known, medicinal chemists would go on to develop much more effective gene-targeted drugs. Unlike most early proponents of the ‘War on Cancer’, who thought that DNA-damaging chemotherapeutic agents would bring real victories in one to two decades, I thought three if not four more decades of focused research would need to pass before we would be in a position to go all out for total victory [1]. In fact, only after the 1988–2003 Human Genome Project provided the world with the highly accurate sequences for three billion human DNA letters has it been possible to begin to approach the true genetic complexity of cancer.

## 2. Molecular pathways to cancer as revealed through DNA sequencing

By now we know that mutations in at least several hundred human genes (out of a total of 21 000 genes) become serious ‘drivers’ of the abnormal cell growth and division process that generates human cancer [2]. They do so because they encode the protein components of ‘signal transduction pathways’ that enable external signals (growth factors) to move from the cell surface receptors to key promoter–enhancer regions along the 24 human chromosomes. There they turn up the expression of genes needed for cell growth and division as well as the evasion of programmed cell death, the latter of which much underlies the ever-growing resistance of late-stage aggressive cancer cells to radio- and chemotherapeutic therapies. Most importantly, there exist multiple molecular pathways that bring about cell growth and proliferation, each with their own specific surface receptors, cytoplasmic transducers, and promoters and enhancers of gene expression [3].

Much potential cross talk exists between these pathways, allowing new DNA mutations to create new pathways to cancer when pre-existing ones are blocked. Already we know that the emergence of resistance to the gene *BRAF*-targeted anti-melanoma drug Zelboraf frequently results from driver pathway cross talk, as does resistance to the targeted drugs Iressa and Tarceva when they are deployed against EGFR-driven lung cancers. Given the seemingly almost intrinsic genetic instability of many late-stage cancers, we should not be surprised when key old timers in cancer genetics doubt being able to truly cure most victims of widespread metastatic cancer.

Resistance to gene-targeted anti-cancer drugs also comes about as a consequence of the radical changes in underlying patterns of gene expression that accompany the epithelial-to-mesenchymal cell transitions (EMTs) that cancer cells undergo when their surrounding environments become hypoxic [4]. EMTs generate free-floating mesenchymal cells whose flexible shapes and still high ATP-generating potential give them the capacity for amoeboid cell-like movements that let them metastasize to other body locations (brain, liver, lungs). Only when they have so moved do most cancers become truly life-threatening.

## 3. Epithelial-to-mesenchymal transitions are a consequence of changes in transcriptional regulation

EMTs leave intact the pre-existing order of DNA bases while changing the way they are read into RNA transcripts. Underlying transcriptional regulation are site-specific DNA-binding proteins, and sometimes regulatory RNAs, that recruit to genes the machinery required to read those genes. This includes the general transcription machinery and also enzymes that modify the histones around which chromosomal DNA is wound, and the DNA itself. These enzymes mediate methylation and acetylation of histones, as well as remodelling of the nucleosomes in various ways, and methylation of DNA bases, changes that can influence how a given gene is expressed. Regulation of transcription extends far beyond its role in influencing how cancer cells respond to changes in their environmental surroundings. This regulation underlies all the multiple switches that accompany the transition of fertilized eggs into the differentiated cells (lung, kidney, etc.) of mature organisms.

## 4. IL6-like cytokines drive mesenchymal cells to commence cell proliferation

Much holding back the creation of effective drugs against mesenchymal cancer cells has long been ignorance of the externally driven signalling pathways propelling them into stem cell growth and subsequent differentiation. Most attention until now has been focused on the Wnt signalling pathway that sends β-catenin into the cell nucleus to activate the TCF transcription factor for essential roles in EMTs as well as stem cell functioning [5,6]. An even more important villain may have been virtually staring in our faces for almost two decades—one or more of the cytokine mediators of inflammation and immunity, in particular, the IL6 interleukin. IL6 blood serum levels, for example, steadily go up as incurable cancers become more life-threatening [7,8]. Autocrine loops probably exist where cytokine binding to their respective cell surface receptors sets into motion downstream gene-activating pathways that not only generate more IL6 molecules but give their respective cancer cells an aura of almost true immortality by blocking the major pathway to programmed cell death (apoptosis). *Pushing by cytokines of otherwise quiescent mesenchymal cancer cells to grow and divide probably explains why anti-inflammatory agents such as aspirin lead to much less cancer in those human beings who regularly take them* [9].

Unfortunately, the inherently very large number of proteins whose expression goes either up or down as the mesenchymal cancer cells move out of quiescent states into the cell cycle makes it still very tricky to know, beyond the cytokines, what other driver proteins to focus on for drug development. Ideally, we should largely focus first on finding inhibitors of cancer cell proliferation as opposed to inhibitors of cancer cell growth. Inhibiting, say, the synthesis of cellular molecular building blocks will slow down not only the metabolism of cancer cells but also that of our body's normally functioning cells. By contrast, blocking proteins specifically moving through the cell cycle should leave untouched the normal functioning of the vast majority of our body's cells and so generate much less unwanted side effects.

## 5. The gene transcription activator Myc allows cells to move through the cell cycle

Long thought to be a key, if not *the* key, protein against which to develop cell-proliferation-inhibiting drugs is the powerful gene transcription activator Myc. First known for its role in driving cancers of blood-forming lymphocytes (e.g. Burkitt's lymphoma), Myc now also has been found to be a key driver of the rapidly fatal ‘small cell’ lung cancers as well as the likely driver of many late-stage incurable cancers, including receptor negative and ductal breast cancers. *Lots of* *Myc* *may turn out to be an essential feature of much of the truly incurable cancer*. It simultaneously turns up the synthesis of the more than 1000 different proteins required to move all cells through the cell cycle. Although precisely how this almost 400-amino acid long polypeptide works at the molecular level remains to be worked out, it seems to play a unique role that cannot be handled by any other class of transcription factors. Unlike our first hunch that Myc was somehow an on–off specifier of gene activity, it is a nonlinear amplifier of expression acting universally on active genes except for the immediate early genes that become expressed before *Myc* [10,11]. Already many serious efforts have been made to develop drugs that block its cell-proliferation-promoting activities. Unfortunately, all such direct efforts have so far failed.

Using a dominant negative plasmid that blocks all Myc functions, Gerard Evans’ laboratory, first at UCSF and now in Cambridge, UK, has used mouse xenograph models of several major human cancers to show Myc's indispensable role in moving through the cell cycle [12]. Although mouse stem cells in Myc's absence stop growing and dividing, they resume normal functioning when *Myc* is turned back on. By contrast, the turning off of *Myc* in human cancer cells preferentially drives them into programmed cell death (apoptosis) with one important exception: pancreatic adenocarcinoma cells do not enter into apoptosis, quite possibly explaining why pancreatic cancer is so resistant to virtually all cell-killing reagents (G. Evans 2012, personal communication).

## 6. Bromodomain 4 proteins play essential roles in maintaining the Myc levels necessary for leukaemic cell growth and division

An unanticipated powerful way for lowering Myc levels in haematopoietic cancers has emerged from the discovery that the incurable nature of *MLL-AF9* acute myeloid leukaemia (AML) depends upon the presence of the not yet well understood protein bromodomain 4 (BRD4). When JQ1, developed last year to treat the BRD4-driven rare *NUT* midline carcinoma, was used on human *MLL-AF9* AML cells, they rapidly stopped multiplying and differentiated into macrophages [13,14]. At the same time, Myc levels rapidly plunged. Most importantly, JQ1 does not block the normal macrophage production, suggesting that Myc levels in macrophage-forming stem cells do not depend upon BRD4. Their formation must depend on a different chromosomal remodeller.

## 7. *Myc* is turned on through multiple molecular pathways

How *Myc* is turned on not only in other cancers but also during normal human development remains largely to be worked out. Likewise not known is how the BRD4 protein at the molecular level helps turn on Myc synthesis in MLL-AF9-driven leukaemia. Until JQ1 goes into the clinic against leukaemia late this year, we will not moreover know for sure whether resistance to JQ1 will compromise its clinical utility. Unfortunately, the answer is probably yes because artificially turning up *Myc* by means that bypass *BRD4* causes JQ1 resistance. Moreover, there are already known multiple ways to turn on *Myc* expression in normal cells, each starting by signals binding to specific cell surface receptors then moving through one or more layers of signal transducers to the nucleus to turn up the transcription of genes needed for cell growth and division. Myc synthesis is not only downstream of the cytokine Jak–Stat3 signal transduction pathway but also downstream of the HER2–RAS–RAF–SHp2–ERK3 pathway that helps drive the growth of much, if not most, breast cancer [15]. Whether they in turn feed into BRD protein-dependent gene-activating pathways remains for the future to reveal. A multiplicity of Myc-inhibiting specific drugs may have to be in our arsenal before we can routinely move beyond delaying death from incurable cancers to true lifetime long cures.

## 8. Detecting key cancer cell vulnerabilities through RNAi screens

That the BRD4 protein is among the major Achilles' heels of incurable AML became known not because of a chance observation but by using a powerful new methodology for detecting molecular weaknesses that are cancer cell-specific. At its heart has been the deployment over the past several years by Greg Hannon at Cold Spring Harbor Laboratory of short hairpin RNA molecules (shRNAs) specifically designed to knock back the functioning of single human genes [16]. A genome shRNA library containing multiple probes (four to six) for each human gene possesses some 100 000 shRNAs. Testing all of them extensively against just one type of cancer still poses a formidable, logistical challenge likely to require 1- to 2-year long intervals for even ‘big science laboratories’.

Much smaller highly focused libraries, however, now can be deployed by high-quality, university-level science laboratories provided there already exist hints as to what molecular vulnerabilities might be found. Forearmed by knowledge that invariably incurable forms of acute myeloblastic leukaemia (AML) originate from rearrangements of a key gene involved in epigenetic chromosomal remodelling, Chris Vakoc and Johannes Zuber at the Cold Spring Harbor Laboratory found the gene-activating BRD4 as the most pronounced potential molecular weakness of an *MLL-AF9* human AML. They did so by screening libraries of only some 1000 probes designed to knockout 234 genes coding for the key proteins involved in epigenetic-driven gene expression.

Most recently, Vakoc has found three other major protein players (Menin, Ezh1/2 and Eed) that work together with BRD4 to make *MLL-AF9* AML incurable by currently deployed anti-cancer drugs [17]. Drugs inhibiting their respective functioning should also provide effective anti-AML agents. *Ezh1/2* and *Eed* code for polycomb proteins that block specific gene expression, whereas the *Menin* gene, like the *BRD4* gene, activates gene expression. Loss of functional Ezh1/2 and Eed blocks the expression of the *CdKn2a* gene-encoded p16 and p19 proteins that have widespread cell-cycle-progression-blocking roles. The Menin protein's molecular role probably involves its already known binding to MLL. Like BRD4, it may have a Myc-level-raising role. Finding out how such chromosome remodelling dependencies emerge and evolve during tumour progression will directly impact the clinical implementation of epigenetic-based anti-cancer therapies.

## 9. BRD4 functioning is vital not only for fast-growing leukaemias but also for many, if not most, dangerous lymphomas and myelomas

As soon as possible, we must find out in more detail how far the drug JQ1's anti-cancer actions extend beyond *MLL-AF9*-specific AMLs. Already we know that in mice it stops equally well the more curable, non-*MLL* rearranged strains of AML as well as all forms of acute lymphocytic leukaemia (ALL). BRD4's capacity to heighten Myc levels thus probably extends over almost all leukaemias. Whether the polycomb proteins of ALL, like those of AML, also turn off the cell-cycle-inhibiting *CdKn2a*-coded proteins p16 and p19 remains to be seen. JQ1 also stops the growth in mice of many fast-growing B-, and T-cell lymphomas, suggesting that their untreated BRD4 protein maintains their high Myc levels necessary to make them fatal. In JQ1-resistant lymphomas (e.g. Jurkat cell), Myc synthesis must be turned on by a different route. Cell lines from most human multiple myeloma victims also frequently show high sensitivity to JQ1 [18]. There, the twosome cocktail of JQ1 and the now widely deployed proteasome inhibitor Velcade reinforce each other's anti-myeloma actions. When JQ1 becomes broadly available clinically, hopefully by mid-2013, it may considerably lengthen the 3–5 more years of additional life provided to most myeloma victims by Velcade administration.

JQ1 also significantly slows down the growth of a small but real number of cell lines derived from many major solid cancers (e.g. prostate and melanoma). BRD4 may have been only called into play late as these cancers evolve to become more aggressive. Of more importance is JQ1's failure to stop the growth of the vast majority of solid tumour cell lines. The heightened Myc levels needed by, say, cancers of the prostate and breast may instead be provided by the intervention of one or more of the some 35 other BRD proteins or other chromatin regulators. Unfortunately, we do not yet know how the vast majority of them function beyond the fact that their BRD pockets, by binding to the acetyl groups, help turn on, not turn off, gene activation. JQ1's unanticipated blocking of sperm functioning most excitingly has led to the recent discovery of a testis-specific bromodomain (BRDT) essential for chromatin remodelling during spermatogenesis. Occupancy of the BRDT acetyl-lysine pocket by JQ1 generates a complete and reversible contraceptive effect [19]. Early evidence suggests that BRDT does not promote Myc synthesis. There may be out there soon to be found, say, breast-specific or prostate-specific *BRD* gene activators. Most important to learn is whether they also do or do not drive Myc synthesis.

## 10. The circadian rhythm regulator (PER2) by negatively regulating Myc levels functions as an important tumour suppressor

Myc's paramount role in moving cancer cells through the cell cycle has recently been reinforced by two highly independent RNAi screens to find genes whose loss of function selectively kills cancer cells [20,21]. In sampling largely different sets of genes, they both honed in on the gene *CSNKe* coding for protein kinase casein kinase 2 epsilon. Among its many multiple targets for phosphorylation and subsequent proteosome-mediated degradation is the transcription factor *PER2* gene whose selective binding to DNA turns off the function of many genes including *Myc*. Already long known has been PERIOD 2 (PER2) involvement as a clock protein at the heart of the circadian rhythms of higher animal cells. Later, quite unexpectedly, PER2 was found to function as a tumour suppressor, with the absence of both its copies causing the rate of radiation-induced cancers to rise. It now seems obvious that its anti-cancer action arises from its ability to turn off *Myc*. In PER2's absence, Myc levels greatly rise, thereby explaining why tumours of many types all display higher levels of CSNKe than found in their normal cell equivalents. Common sense suggests that specific CSNKe inhibitors should soon be broadly tested against a large variety of human cancers.

## 11. High-Myc-driven, fast proliferating cells possess cell cycle vulnerabilities

High-Myc-level proliferating cells less efficiently proceed through the mitotic cycle than cells driven by lower Myc levels. Why high Myc leads to many more mitotic-generated chromosome abnormalities has recently been explained through a large RNAi screen designed to reveal ‘synthetic lethal’ genes that only have vital function under conditions of high Myc. Most unexpectedly, they pinpointed key roles for the SUMO-activating genes *SAE1* and *SAE2* involved in proteasome-specific protein degradation [22]. When they are blocked from functioning, large numbers of Myc-driven genes somehow become switched from on to off. As expected, many function in the formation and breakdown of the mitotic spindle. A much less anticipated second class functions in ubiquitin-based, proteasome-mediated protein degradation. Conceivably, the fast growth rates of high-Myc-level-driven proliferating cells generate more mitosis-involved proteins than their respective proteasomes can timely breakdown. In any case, drugs designed to block *SAE1* and *SAE2* should preferentially kill fast-proliferating cancer cells.

High-Myc-level vulnerability is also generated by suboptimal supplies of CD kinase 1 (CDK1) that functions with the A type cyclins during the late S phase of the cell cycle. As long as the Myc levels are those of normal cells, proliferating cells have sufficient CDK1. But when more Myc leads to faster cell cycles, much more CDK1 is required to prevent failed cell divisions. So, it makes a prime candidate for the development of an effective drug against high-Myc-driven cancers [23].

## 12. Selectively killing cancer cells through exploiting cancer-specific metabolic and oxidative weaknesses

We must focus much, much more on the wide range of metabolic and oxidative vulnerabilities that arise as consequences of the uncontrolled growth and proliferation capacities of cancer cells. As human cancers become driven to more aggressive glycolytic states, their ever-increasing metabolic stress makes them especially vulnerable to sudden lowering of their vital ATP energy supplies. 3-Bromopyruvate, the powerful dual inhibitor of hexokinase as well as oxidative phosphorylation, kills highly dangerous hepatocellular carcinoma cells more than 10 times faster than the more resilient normal liver cells and so has the capacity to truly cure, at least in rats, an otherwise highly incurable cancer [24,25]. The structurally very different hexokinase inhibitor 2-deoxyglucose, through its ability to block glycolysis, also has the potential for being an important anti-cancer drug. Not surprisingly, it works even better when combined with inhibitors of ATP-generating oxidative phosphorylation such as the mitochondrial target drug Mito Q [26].

A key mediator of cellular response to falling ATP levels is the AMP-dependent protein kinase AMPK, which in times of nutritional stress phosphorylates key target proteins to push metabolism away from anabolic growth patterns [27]. By inhibiting mTOR it slows protein synthesis, and by phosphorylating acetyl-CoA carboxylase it slows down lipid synthesis. The glycolytic pathways that produce the cellular building blocks are indirectly controlled by AMPK through its phosphorylation of the p53 transcription factor. Activated p53 slows down glycolysis during cell cycle arrest through turning on its *TIGAR* gene target. Its respective protein breaks down the key regulator of glycolysis fructose 2,6-bisphosphate as well as blocking further cell cycles through turning on the *p21* gene.

## 13. Preferential cancer cell killing by apoptosis reflects high p53 levels

The enhanced apoptosis capability of early-stage epithelial cancer cells, in comparison with their normal cell equivalents, reflects their higher content of activated p53 transcription factor. Overexpression and amplification of the p53 repressors MDM2 and MDM4 are common across cancer types. In the case of melanomas, p53 function is commonly shut down by overexpression of MDM4. Already a drug exists that through its inhibition of MDM4 makes melanoma much more treatable [28]. Knowing more about why p53 activation sometimes leads to cell cycle arrest (senescence) and under different circumstances results in apoptosis remains an important challenge for the immediate future.

## 14. P53 induces apoptosis by turning on the synthesis of genes whose primary function is the synthesis of reactive oxygen species

How p53 turns on apoptosis was first revealed through elegant gene expression studies carried out in Bert Vogelstein's Johns Hopkins laboratory in 1997 [29]. Although looking for genes expressed only during apoptosis, they discovered a set of 13 p53-induced genes (*PIG* genes), each of which are likely key players in the cellular synthesis of reactive oxygen species (ROS; H_2_O_2_ hydrogen peroxide, the OH^−^ radiation and O_2_^−^ superoxides). *PIG3*, for example, codes for a quinone oxidoreductase that is a potent generator of ROS [30,31]. p53 target genes also play major roles in downstream processes through turning on synthesis of some 10 different mitochondrial functioning proteins such as BAX, PUMA and NOXA, as well as death receptors such as DR4 and DR5, that in ways yet to be elucidated help carry out the many successive proteolysis stages in apoptosis [32].

Equally important, p53 turns on the synthesis of the key proteins involved in the apoptotic (programmed cell death) elimination of cells that have no long-term future, say, through unsustainable metabolic stress or damage to cellular chromosomes brought about by exposure to ultraviolet or ionizing radiation. So, removing such cells are complex sets of largely mitochondrial-sited degradation events. As the successive stages in apoptosis unravel, the respective dying cells lose mitochondrial functioning and release cytochrome c, culminating in DNA-liberating cell dissolution.

## 15. Leakage from drug-impaired mitochondrial electron transport chains raises reactive oxygen species levels

The mitochondrial electron transport generation of ATP and heat is obligatorily accompanied by the production of ROS (such as the OH^−^ radical, H_2_O_2_ and O_2_^−^ superoxides). Normally, preventing ROS molecules from irreversibly damaging key nucleic acid and protein molecules are potent antioxidative molecules such as glutathione and thioredoxin [33]. When present in normal amounts, they cannot handle the much larger amount of ROS generated when oxidative phosphorylation becomes inhibited by mitochondrial-specific drugs such as rotenone that block feeding of NADH into the respiratory chain or by 3′-3′ diindolylmethane (DIM), the active component in the long-reputed chemo-preventative *Brassica* vegetables, which inhibits the mitochondrial F1F0 ATP synthesis complex [34]. Still-remaining ROS molecules through oxidizing intra-mitochondrial targets induce the apoptotic elimination of cells damaged from excessive oxidative stress. Already, DIM is used as an adjuvant therapy for recurrent respiratory papillomatosis in humans. The molecular mechanism(s) through which ROS induce apoptosis remains to be found—hopefully soon. Now, we will be surprised if they do not somehow directly oxidize and so activate one or more of the BAX-like proteins involved in p53-mediated apoptosis.

That ROS by themselves can mediate apoptosis was recently convincingly shown by the finding that the ‘first-in-class’ anti-cancer mitochondrial drug elesclomol (discovered by Synta Pharmaceuticals through screening for anti-apoptotic agents) kills cancer cells through promoting ROS generation [35]. When these resulting ROS molecules are destroyed through the simultaneous administration of the antioxidant molecule *N*-acetylcysteine, preferential killing of cancer cells stops. The failure of elesclomol to generate apoptosis in non-cancerous cells probably arises from the inherently lower ROS level generated by normal mitochondrial electron transport machinery.

## 16. Reactive oxygen species may directly induce most apoptosis

That elesclomol promotes apoptosis through ROS generation raises the question whether much more, if not most, programmed cell death caused by anti-cancer therapies is also ROS-induced. Long puzzling has been why the highly oxygen sensitive ‘hypoxia-inducible transcription factor’ HIF1α is inactivated by both the, until now thought very differently acting, ‘microtubule binding’ anti-cancer taxanes such as paclitaxel and the anti-cancer DNA intercalating topoisomerases such as topotecan or doxorubicin, as well as by frame-shifting mutagens such as acriflavine [36,37]. All these seemingly unrelated facts finally make sense by postulating that not only does ionizing radiation produce apoptosis through ROS but also today's most effective anti-cancer chemotherapeutic agents as well as the most efficient frame-shifting mutagens induce apoptosis through generating the synthesis of ROS [38–40]. That the taxane paclitaxel generates ROS through its binding to DNA became known from experiments showing that its relative effectiveness against cancer cell lines of widely different sensitivity is inversely correlated with their respective antioxidant capacity [41,42]. A common ROS-mediated way through which almost all anti-cancer agents induce apoptosis explains why cancers that become resistant to chemotherapeutic control become equally resistant to ionizing radiotherapy.

Recent use of a 50 000 member chemical library at MIT's Koch Cancer Center to search out molecules that selectively killed *K-RAS*-transformed human fibroblasts revealed the piperidine derivation lanperisone [43]. ROS generation underlies its cancer cell killing action. Surprisingly, this already clinically used muscle relaxant induced non-apoptotic cell death in a p53 (++ versus−−) independent manner. When lanperisone was applied in the presence of the ROS-destroying antioxidant scavenger molecules deferoxamine, butylated hydroxylamine or the antioxidant trolox, no activity was observed.

## 17. Blockage of reactive-oxygen-species-driven apoptosis by antioxidants

Although we know ROS as a positive force for life through their apoptosis-inducing role, for much longer we have feared them for their ability to irreversibly damage key proteins and nucleic acid molecules. So when not needed, they are constantly being neutralized by antioxidative proteins such as glutathione, superoxide dismutase, catalase and thioredoxin. Controlling their synthesis as well as that of many more minor antioxidants is the Nrf2 transcription factor, which probably came into existence soon after life as we know it started. Most importantly, at Cancer Research UK in Cambridge, David Tuveson's laboratory has recently shown that Nrf2 synthesis is somehow upregulated by the cell growth and division-promoting *RAS*, *RAF* and *MYC* oncogenes [44]. Biologically, this makes sense because we want antioxidants present when DNA functions to make more of itself.

The fact that cancer cells largely driven by RAS and Myc are among the most difficult to treat may thus often be due to their high levels of ROS-destroying antioxidants. Whether their high antioxidative level totally explains the effective incurability of pancreatic cancer remains to be shown. The fact that late-stage cancers frequently have multiple copies of *RAS* and *MYC* oncogenes strongly hints that their general incurability more than occasionally arises from high antioxidant levels. Clearly important to learn is what other molecules exist that turn on Nrf2 expression. During the yeast life cycle and probably that of most organisms, oxidative phosphorylation is clearly separated by time from when DNA synthesis occurs. Whether Nrf2 levels also go up and down during the cell cycle remains important to be known soon.

## 18. Enhancing apoptotic killing using pre-existing drugs that lower antioxidant levels

Already there exist experiments with haematopoietic cells in which the cancer-cell-killing capacity of the ROS generator arsenic trioxide (As_2_O_3_) has been shown to be inversely correlated with the content levels of the major cellular antioxidant glutathione [45]. As_2_O_3_ also knocks down the reductive power of thioredoxin necessary for several key steps in cellular metabolism. Its capacity to inhibit both thioredoxin and glutathione widens its potential for a successful deployment against many major cancers beyond promyeloblastic leukaemia. Also capable of enhancing the cytotoxic effect of As_2_O_3_ is ascorbic acid, which, though known for its antioxidant role in cells, is converted into its oxidizing form dehydroascorbic acid. Unfortunately, up until now, we do not yet have clinically effective ways to lower glutathione levels. Lowering its level through deployment of the drug buthionine sulphazine that blocks its synthesis leads quickly to upregulation of the Nrf2 transcription factor that in turn upregulates glutathione synthesis [46]. A more general way to reduce antioxidant levels deploys motexafin gadolinium, a member of a class of porphyrin molecules called texaphyrins. Through a process called futile redox recycling, it transfers hydrogen from antioxidants to produce ROS. Unfortunately, clinical trials designed to show its enhancement of chemo- and radiotherapies have so far shown only modest life extensions as opposed to cures.

Through selecting for compounds that preferentially induce apoptosis in cancer cells as opposed to normal cells, the natural product piperlongumine from the *Piper longum* plant was recently revealed as a potential anti-cancer drug [47]. Most exciting, it mediates its action through its binding to the active sites of several key cellular antioxidants (e.g. glutathione *S* transferase and carbonyl reductase 1) known to participate in cellular responses to ROS-induced oxidative stress. That piperlongumine failed to raise ROS levels in non-cancerous cells probably resulted from their inherently lower levels of these antioxidants which, in turn, result from less activation of the Nrf2 transcription factor.

## 19. Anti-angiogenic drugs work only when used in conjunction with reactive oxygen species generators

The non-toxic anti-angiogenesis protein endostatin (discovered and promoted in the late 1990s in Judah Folkman's Boston laboratory and now resurrected by Yongzhang Luo in Beijing) shows anti-cancer activity only when it is used together with conventional chemotherapeutic agents. This fact, long puzzling to me, may be due to the chemotherapeutic component providing the ROS needed for cancer cell killing [48]. By itself, the hypoxia resulting from endostatin action may not be sufficient for cancer cell killing. A similar explanation may explain why Genentech's avastin also only works when combined with chemotherapy. By contrast, the killing of mutant *BRAF* melanoma cells by Zelboraf works very well in the absence of any obvious direct source of ROS. Conceivably, the metabolic stress resulting from its turning off the RAS–ERK pathways somehow shuts down the Nrf2 pathways, letting ROS rise to the level needed to kill the drug-weakened melanoma cells.

## 20. Lower reactive oxygen species levels in stem cells reflect higher levels of antioxidants

For more than a decade, there has existed too long ignored evidence that normal stem cells have lower ROS levels than their differentiated progeny. Just a year ago, even more convincing experimentation showed that breast cancer stem cells also contain lower ROS levels than those found in their cancerous epithelial-like progeny cells [49]. All stem cells, be they normal or cancerous, probably have lower ROS levels as a result of their corresponding higher levels of prominent antioxidant molecules such as glutathione and thioredoxin. Most likely, these heightened amounts have evolved to protect chromosomal RNA from ROS-induced damage to the more exposed region of chromosomal DNA as it undergoes changes in compaction as it moves through the cell cycle. Whether all dividing cells have higher antioxidant levels remains to be worked out. If so, all stem cells will be inherently much more resistant to ROS-induced apoptotic killing than more differentiated, much less antioxidant-rich progeny cells.

## 21. Metformin selectively targets (kills) mesenchymal cancer stem cells

Already we have at our disposal a relatively non-toxic, excessively well-tested drug that preferentially kills mesenchymal stem cells. In a still much unappreciated article published three years ago in *Cancer Research*, Kevin Struhl's laboratory at Harvard Medical School first showed that metformin, a blocker of stage 2 oxidative phosphorylation, selectively targets stem cells. When so applied with chemotherapeutic agents to block xenographic tumour growth, it induces prolonged remission if not real cures [50,51]. But when metformin was left out of these experiments, subsequent multiplication of unkillable mesenchymal stem cells lets these xenographs grow into life-threatening forms, showing that chemotherapy by itself does not kill stem cells. This most widely used anti-diabetic drug's heightened ability to kill late-stage mesenchymal cancer cells probably explains why those humans who use it regularly have reduced incidences of many cancers.

Metformin is presently being added to a number of anti-cancer chemotherapeutic regimes to see whether it magnifies their effectiveness in humans. The fact that metformin works much more effectively against p53**^− −^** cells suggests that it may be most active against late-stage cancers, the vast majority of whose cells have lost both of their *p53* genes. By contrast, the highly chemo-radio-sensitive early-stage cancers against which most of anti-cancer drug development has focused might very well show little metformin effectiveness. By the end of 2013, we should know whether it radically improves any current therapies now in use. Highly focused new drug development should be initiated towards finding compounds beyond metformin that selectively kill stem cells. And the reason why metformin preferentially kills p53**^− −^** stem cells should be even more actively sought out.

## 22. Free-radical-destroying antioxidative nutritional supplements may have caused more cancers than they have prevented

For as long as I have been focused on the understanding and curing of cancer (I taught a course on Cancer at Harvard in the autumn of 1959), well-intentioned individuals have been consuming antioxidative nutritional supplements as cancer preventatives if not actual therapies. The past, most prominent scientific proponent of their value was the great Caltech chemist, Linus Pauling, who near the end of his illustrious career wrote a book with Ewan Cameron in 1979, *Cancer and Vitamin C*, about vitamin C's great potential as an anti-cancer agent [52]. At the time of his death from prostate cancer in 1994, at the age of 93, Linus was taking 12 g of vitamin C every day. In light of the recent data strongly hinting that much of late-stage cancer's untreatability may arise from its possession of too many antioxidants, the time has come to seriously ask whether antioxidant use much more likely causes than prevents cancer.

All in all, the by now vast number of nutritional intervention trials using the antioxidants β-carotene, vitamin A, vitamin C, vitamin E and selenium have shown no obvious effectiveness in preventing gastrointestinal cancer nor in lengthening mortality [53]. In fact, they seem to slightly shorten the lives of those who take them. Future data may, in fact, show that antioxidant use, particularly that of vitamin E, leads to a small number of cancers that would not have come into existence but for antioxidant supplementation. Blueberries best be eaten because they taste good, not because their consumption will lead to less cancer.

## 23. A much faster timetable for developing anti-metastatic drugs

The world of Physics already knew 20 years ago that it had no choice but to go very big for the Higgs boson. To the civilized world's great relief, they now finally have it. Biology and Medicine must likewise now again aim big—as when we first promised the world in 1988 that the still to be found human genome would later prove indispensable for the curing of most cancers and so went for it big. If, however, we continue to move forward at today's never frantic, largely five-day working week, the never receding 10–20 year away final victory that our cancer world now feels safe to project will continue to sink the stomachs of informed cancer victims and their families. That we now have no General of influence, much less power, say an Eisenhower or even better a Patton, leading our country's War on Cancer says everything. Needed soon is a leader that has our cancer drug development world working every day and all through the night.

The now much-touted genome-based personal cancer therapies may turn out to be much less important tools for future medicine than the newspapers of today lead us to hope [54]. Sending more government cancer monies towards innovative, anti-metastatic drug development to appropriate high-quality academic institutions would better use National Cancer Institute's (NCI) monies than the large sums spent now testing drugs for which we have little hope of true breakthroughs. The biggest obstacle today to moving forward effectively towards a true *war against cancer* may, in fact, come from the inherently conservative nature of today's cancer research establishments. They still are too closely wedded to moving forward with cocktails of drugs targeted against the growth promoting molecules (such as HER2, RAS, RAF, MEK, ERK, PI3K, AKT and mTOR) of signal transduction pathways instead of against Myc molecules that specifically promote the cell cycle.

Most needed now are many new anti-Myc drugs beyond the exciting new BRD4 inhibitors, such as JQ1, as well as multiple drugs that inhibit the antioxidative molecules that likely make, say, pancreatic cancer so incurable. They should much enhance the effectiveness of all current radio- and chemotherapeutic regimes. As such, they will likely cure many more now incurable cancers. How they will interact as cocktail partners with the newer targeted therapies that do not directly generate ROS remains to be seen. Equally important may be an expanded search for drugs that prevent p53 breakdown.

## 24. A billion dollars should suffice to identify all the remaining proteins needed for curing most metastatic cancer

The total sum of money required for RNAi methodologies to reveal the remaining major molecular targets for future anti-cancer drug development need not be more than 500–1000 million dollars. Unfortunately, the NCI now is unlikely to take on still one more big science project when it is so hard-pressed to fund currently funded cancer programmes. Still dominating NCI's big science budget is The Cancer Genome Atlas (TCGA) project, which by its very nature finds only cancer cell drivers as opposed to vulnerabilities (synthetic lethals). While I initially supported TCGA getting big monies, I no longer do so. Further 100 million dollar annual injections so spent are not likely to produce the truly breakthrough drugs that we now so desperately need.

Happily, the first RNAi whole genome big screen backed by a ‘big pharma’ firm has just started with Pfizer working with the Cold Spring Harbor Laboratory. Even, however, if several more giants working separately join in, collectively they will naturally focus on major cancers such as those of the breast, colon and lung. I doubt they will soon go big against, say, either melanoma or oesophageal cancer. Greg Hannon here at Cold Spring Harbor will probably be the first academic scientist to come to grips with the non-trivial experimental challenges provided by whole genome, 100 000 RNAi probe screens, through both his collaboration with Pfizer and through using monies separately provided by the Long Island-based Lustgarten Foundation's support for a comprehensive pancreatic cancer target screen and by Hollywood's ‘stand up against cancer’ support for breast cancer drug target identification. Although our enthusiasm for big RNAi screens remains far from universally shared, lack of money should not now keep us from soon seeing whether whole genome methodologies live up to their much-touted expectations [55]. The Cold Spring Harbor Laboratory happily has the means to move forward almost as if it were in a true war.

Further financial backing, allowing many more cancer-focused academic institutions to also go big using RNAi-based target discovery as well as to let them go on to the early stages of subsequent drug discovery, is not beyond the might of the world's major government research funding bodies nor that of our world's many, many super billionaires. The main factor holding us back from overcoming most of metastatic cancer over the next decade may soon no longer be lack of knowledge but our world's increasing failure to intelligently direct its ‘monetary might’ towards more human-society-benefiting directions.
